# Characterization of the Inhibitory Effect of Gastrodigenin and Gastrodin on M-type K^+^ Currents in Pituitary Cells and Hippocampal Neurons

**DOI:** 10.3390/ijms21010117

**Published:** 2019-12-23

**Authors:** Chih-Sheng Yang, Ming-Chi Lai, Ping-Yen Liu, Yi-Ching Lo, Chin-Wei Huang, Sheng-Nan Wu

**Affiliations:** 1 Department of Neurology, Taichung Tzu Chi Hospital, Buddhist Tzu Chi Medical Foundation, Taichung City 42743, Taiwan; yashchun@gmail.com; 2 Department of Pediatrics, Chi-Mei Medical Center, Tainan City 71004, Taiwan; vickylai621@gmail.com; 3 Department of Cardiology, National Cheng Kung University Hospital, College of Medicine, National Cheng Kung University, Tainan City 70101, Taiwan; larry@mail.ncku.edu.tw; 4 Department of Pharmacology, College of Medicine, Kaohsiung Medical University, Kaohsiung City 80708, Taiwan; yichlo@kmu.edu.tw; 5 Department of Neurology, National Cheng Kung University Hospital, College of Medicine, National Cheng Kung University, Tainan City 70101, Taiwan; 6 Department of Physiology, National Cheng Kung University Medical College, Tainan City 70101, Taiwan; 7 Institute of Basic Medical Sciences, National Cheng Kung University Medical College, Tainan City 70101, Taiwan; 8 Department of Medical Research, China Medical University Hospital, China Medical University, Taichung 40402, Taiwan

**Keywords:** gastrodigenin, p-hydroxybenzyl alcohol, gastrodin, M-type K^+^ current, L-type Ca^2+^ current and voltage-gated Na^+^ current

## Abstract

Gastrodigenin (HBA) and gastrodin (GAS) are phenolic ingredients found in Gastrodia elata Blume (GEB), a traditional Chinese herbal medicine. These compounds have been previously used to treat cognitive dysfunction, convulsion, and dizziness. However, at present, there is no available information regarding their potential ionic effects in electrically excitable cells. In the current study, the possible effects of HBA and GAS on different ionic currents in pituitary GH3 cells and hippocampal mHippoE-14 neurons were investigated using the patch-clamp technique. The addition of HBA or GAS resulted in the differential inhibition of the M-type K^+^ current (*I*_K(M)_) density in a concentration-dependent manner in GH_3_ cells. HBA resulted in a slowing of the activation time course of *I*_K(M)_, while GAS elevated it. HBA also mildly suppressed the density of erg-mediated or the delayed-rectifier K^+^ current in GH_3_ cells. Neither GAS nor HBA (10 µM) modified the voltage-gated Na^+^ current density, although they suppressed the L-type Ca^2+^ current density at the same concentration. In hippocampal mHippoE-14 neurons, HBA was effective at inhibiting *I*_K(M)_ density as well as slowing the activation time course. Taken together, the present study provided the first evidence that HBA or GAS could act on cellular mechanisms, and could therefore potentially have a functional influence in various neurologic disorders.

## 1. Introduction

Gastrodigenin (p-hydroxybenzyl alcohol, HBA) is a phenolic compound found in traditional Chinese herbal medicine Gastrodia elata Blume (GEB), which is commonly known as Tian Ma in Chinese or Chunma in Korean [1,2]. Gastrodin (GAS), the glucoside of HBA, is the major bioactive ingredient that can be isolated from GEB. In East Asian countries, the dried rhizomes of GEB have been widely used for centuries to treat many neurological or psychiatric disorders including convulsive disorders, dizziness, dementia, depression, and migraines [3]. Increasing research is being conducted to determine the underlying molecular mechanisms through which the active constituents of GEB may exert their pharmaceutical effects.

GAS has been demonstrated to penetrate the blood–brain barrier, and it has been suggested that it may produce neuroprotective effects against hypoxia, oxidative injuries, or glutamate induced excitotoxicity [4,5,6]. Recent studies have also provided evidence of its antiepileptic, anxiolytic, free-radical scavenging, anti-inflammatory, and anti-obesity actions [3,7]. After entering the central nervous system, GAS is metabolized to HBA [8]. HBA is an aglycone derivative of gastrodin. Some previous studies have addressed the potential promoting effects of HBA on cell proliferation and neuroblast differentiation in the dentate gyrus of mice [9]. In addition, HBA has been previously reported to improve learning and memory consolidation [10]. Despite strong evidence of their beneficial effects on various neurological disorders, HBA and GAS have so far been limited with respect to their effects on membrane ion currents in neurons and neuroendocrine cells.

The KCNQ2, KCNQ3, and KCNQ5 genes are known to encode for the core subunits of the K_V_7.2, K_V_7.3, and K_V_7.5 channels, respectively. Increased activity of these K_V_ channels in neurons, or neuroendocrine or endocrine cells, can generate a unique population of the K^+^ current, named the M-type K^+^ current (*I*_K(M)_). This unique type of K^+^ current is characterized by its activation in response to a low threshold voltage and upon activation, it displays slowly activating and deactivating properties [11,12]. The subfamilies of the K^+^ channels regulate outward K^+^ current, maintain resting membrane potential, and control the neuronal firing threshold. Importantly, the targeting of *I*_K(M)_ has been increasingly recognized as an adjunctive regimen for the treatment of different neurological disorders that are linked to neuronal excitability such as cognitive dysfunction, neuropathic pain, and epilepsy [13,14]. However, to the best of our knowledge, exactly how HBA or GAS exerts any effect on the amplitude and gating of this current has thus far been largely unexplored, despite HBA and GAS demonstrating a wide range of biological activities on the central nervous system in both in vivo and in vitro [3,4,5,6,7].

In light of the situation described above, the present study aimed to investigate the possible underlying mechanism of HBA or GAS action on different types of membrane ionic currents (e.g., *I*_K(M)_, *erg*-mediated K^+^ current [*I*_K(erg)_], delayed-rectifier K^+^ current [*I*_K(DR)_], voltage-gated Na^+^ current [*I*_Na_], and L-type Ca^2+^ current [*I*_Ca,L_]) in two different types of electrically excitable central neurons, the pituitary GH_3_ cells and hippocampal mHippoE-14 neurons. Findings from the current study revealed that HBA and GAS can inhibit *I*_K(M)_ in a concentration- and state-dependent manner in the tested cell types. These findings could lead to the further exploration of HBA and GAS pharmacological actions or other structurally similar phenolic compounds.

## 2. Results

### 2.1. Inhibitory Effect of HBA (Gastrodigenin) on I_K(M)_ Density in GH_3_ Cells

**Figure 1:**
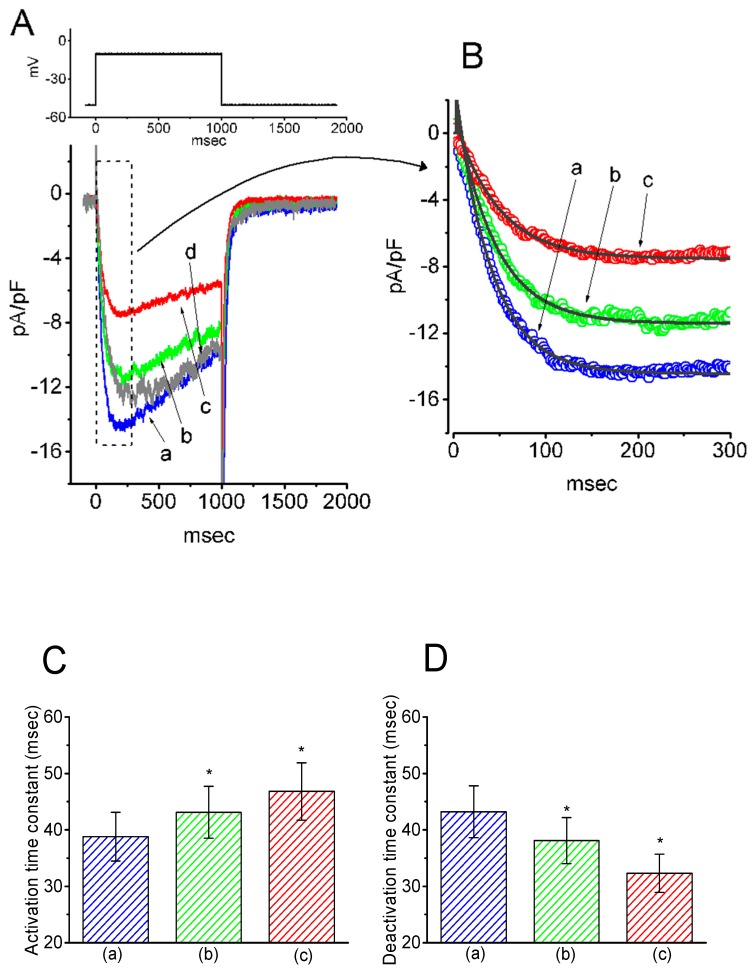
Inhibitory effect of gastrodigenin (HBA) on *I*_K(M)_ density recorded from pituitary GH_3_ cells. As the whole-cell mode was established, the examined cell was depolarized from −50 to −10 mV for 1 s, as indicated in the upper part of (A). (**A**) Original traces of *I*_K(M)_ density obtained with or without the addition of HBA. (a) control; (b) 3 µM HBA; (c) 10 µM HBA; (d) washout of 10 µM HBA. (**B**) Expanded record from the dashed box in (A), indicating the effect of HBA on the activation time course of the *I*_K(M)_-density trajectory elicited by membrane depolarization. The data points (open circles) were reduced by a factor of 10 for clarity. Each current trajectory was least-squares fitted by a single exponential (indicated by the smooth line) with activation time constants (τ_act_) of 38.8 ms (a) in the absence of HBA, 43.0 ms (b) in 3 µM HBA, and 46.8 ms (c) in 10 µM HBA. (**C**) and (**D**) illustrate the summary bar graphs showing the effects of HBA on the τ_act_ and τ_deact_ values of *I*_K(M)_-density trajectory, respectively (mean ± SEM; *n* = 11 for each bar). *p < 0.05 vs. control.

In the initial set of experiments, the presence of *I*_K(M)_ in the pituitary GH_3_ cells was examined to investigate whether HBA could exert any effects on the M-type K^+^ current. GH_3_ cells were bathed in a high-K^+^, Ca^2+^-free solution, and the recording pipette was filled with a K^+^-containing solution. Once the whole-cell mode was firmly achieved, the examined cells were maintained at −50 mV, and a depolarizing pulse of −10 mV with a duration of 1 s was subsequently applied to evoke a unique population of K^+^ currents, namely *I*_K(M)_, as previously identified [15]. As depicted in Figure 1, the presence of HBA caused a progressive decrease in the density of *I*_K(M)_ in these cells in a time- and concentration-dependent manner. For example, within 1 min of exposing the cells to 10 µM HBA, the *I*_K(M)_ density when taken from −50 to −10 mV was significantly decreased from 11.5 ± 3.6 to 5.6 ± 0.9 pA/pF (*n* = 11, *p* < 0.05). After the agent was washed out, the current density returned to 11.2 ± 1.8 pA/pF (*n* = 10, *p* < 0.05). Concomitant with the decreased *I*_K(M)_ density, the activation time course of *I*_K(M)_ density elicited by membrane depolarization slowed down, while there was a progressive increase in the deactivation time course of the current density obtained upon repolarization following membrane depolarization to the holding potential (Figure 1B–D). The addition of HBA (10 µM) significantly increased the activation time constant (τ_act_) of *I*_K(M)_ density from 38.8 ± 4.3 to 46.8 ± 5.1 ms (*n* = 11, *p* < 0.05), while it concomitantly diminished the deactivation time constant (τ_deact_) of the current density from 43.2 ± 4.6 to 32.3 ± 3.4 ms (*n* = 11, *p* < 0.05).

### 2.2. Effect of GAS (Gastrodin) on I_K(M)_ Density in GH_3_ Cells

**Figure 2:**
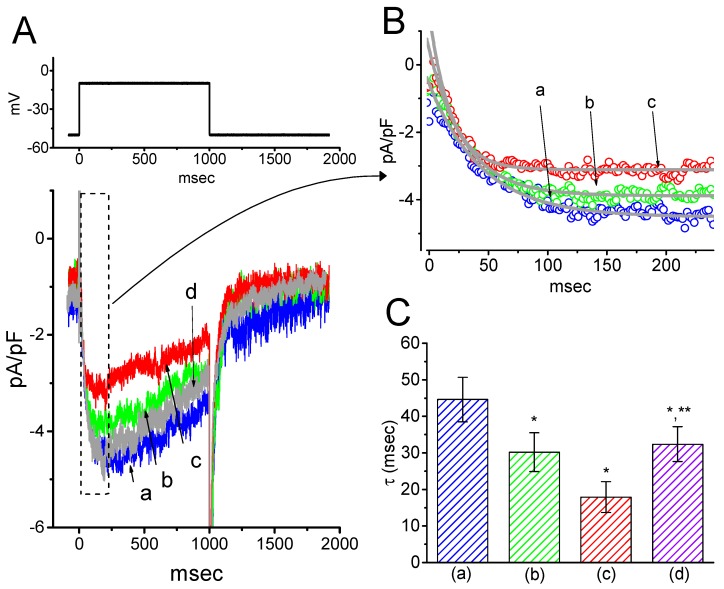
Inhibitory effect of gastrodin (GAS) on *I*_K(M)_ density in GH_3_ cells. (**A**) Original traces of *I*_K(M)_ density obtained with or without the addition of GAS. (a) control; (b) 10 µM GAS; (c) 30 µM GAS; (d) washout of 30 µM GAS. (**B**) Expanded record from the dashed box in (A), indicating the effect of GAS on the activation time course of the *I*_K(M)_-density trajectory as elicited by membrane depolarization. Each current trajectory was least-squares fitted by a single exponential (indicated by the smooth line) with a τ_act_ of 44.6 ms (a) in the absence of GAS, 30.1 ms (b) in 10 µM GAS, and 17.9 ms (c) in 30 µM GAS. (**C**) Summary bar graph showing the effect of GAS and GAS plus flupirtine on the τ_act_ value of *I*_K(M)_-density trajectory in response to membrane depolarization (mean ± SEM; *n* = 9–11 for each bar). (a) control; (b) 10 µM GAS; (c) 30 µM GAS; (d) 30 µM GAS plus 10 µM flupirtine. *p < 0.05 vs. the control. **p < 0.05 vs. (c) 30 µM GAS.

The next series of experiments was designed to test if GAS, a phenolic glucoside that can be metabolized into HBA, could modify the density of *I*_K(M)_ in pituitary GH_3_ cells. The profile of the experimental protocol used was similar to that used for the study of HBA on *I*_K(M)_ density as described above. Notably, as GAS was applied, the density of *I*_K(M)_ in response to membrane depolarization progressively decreased. However, along with the decreased current density, the τ_act_ and τ_deact_ values of *I*_K(M)_ density elicited by membrane depolarization were also reduced (Figure 2). For example, the addition of GAS (30 µM) significantly decreased the *I*_K(M)_ density and τ_act_ to 2.2 ± 0.07 pA/pF and 17.9 ± 4.2 ms (*n* = 11, *p* < 0.05) from the control values of 3.8 ± 0.1 pA/pF and 44.6 ± 6.1 ms, respectively. In the continued presence of 30 µM GAS, the addition of flupirtine (10 µM), a known activator of *I*_K(M)_ [16], attenuated the GAS-induced decrease in both the density and τ_act_ of *I*_K(M)_, as seen in the GH_3_ cells.

### 2.3. Concentration-Dependent Inhibition of I_K(M)_ Density Caused by the Presence of HBA or GAS

**Figure 3:**
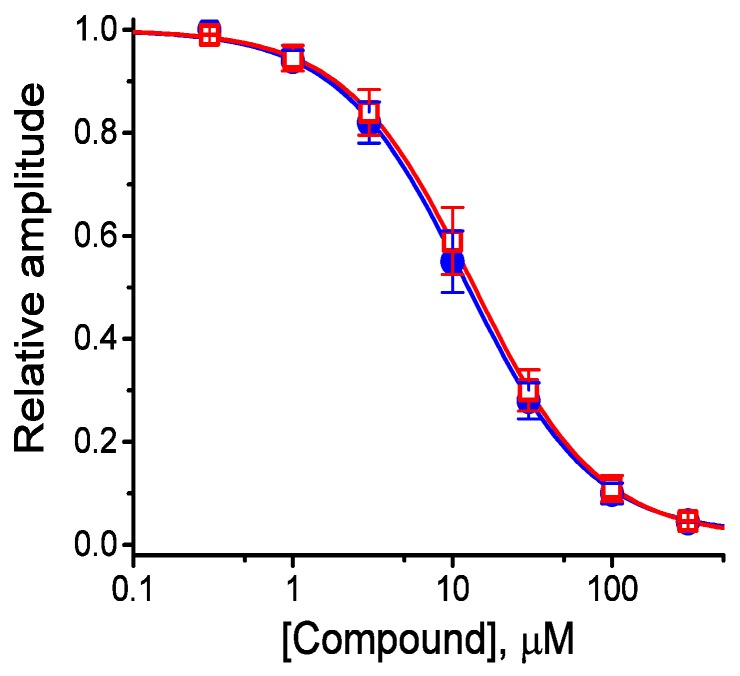
Concentration-response curves for HBA- and GAS-induced inhibition of *I*_K(M)_ amplitude in GH_3_ cells. Each cell was held at −50 mV and depolarizing pulses to −10 mV were applied for 1 s. The amplitude of *I*_K(M)_ measured at the end of each depolarizing pulse from −50 to −10 mV during cell exposure to different concentrations (0.3–100 µM) of HBA or GAS were compared with the control value (mean ± SEM, *n* = 11 for each point). Each smooth line was fitted by a modified Hill function as detailed in the Materials and Methods Section. The values of IC_50_ in the presence of HBA (● blue) and GAS (□ red) were estimated to be 12.1 and 19.4 µM, respectively.

The effects of these agents on *I*_K(M)_ in response to membrane depolarization were further examined and compared in the current study. The concentration-dependent association between the inhibitory effect of HBA or GAS on *I*_K(M)_ as seen in the GH_3_ cells is illustrated in Figure 3. The IC_50_ values of HBA and GAS required for their effect on *I*_K(M)_ amplitude, measured at the end of the depolarizing pulses, were calculated to be 12.1 and 19.4 µM, respectively. Inhibition of *I*_K(M)_ by these compounds had Hill coefficients of 1.1, suggesting that one molecule interacts with a single channel tetramer. The suppressive effect of HBA on *I*_K(M)_ observed in GH_3_ cells tended to be more potent than that of GAS.

### 2.4. Kinetic Study of HBA-induced Blocking of I_K(M)_ Density in GH_3_ Cells

**Figure 4:**
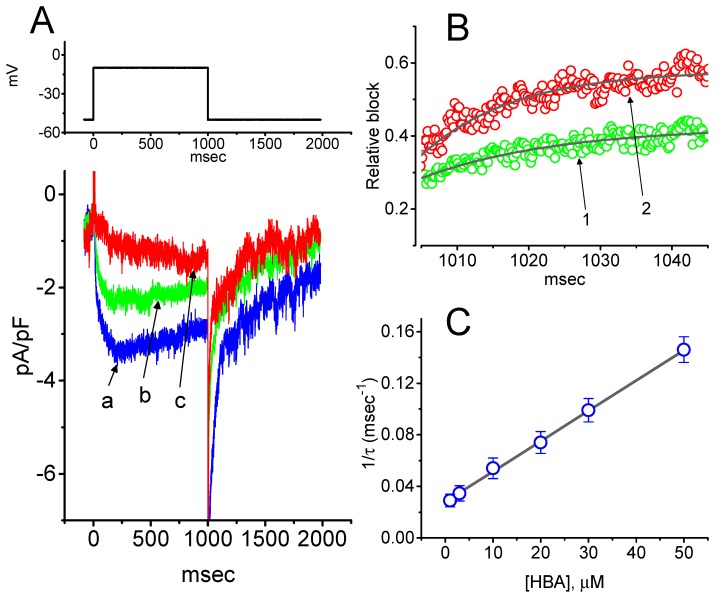
Evaluation of the kinetics of HBA-induced blocking of *I*_K(M)_ density as elicited by membrane depolarization in the GH_3_ cells. The *I*_K(M)_ density established by depolarizing pulses from −50 to −10 mV was measured as cells were exposed to different HBA concentrations (1–50 µM). (**A**) Representative traces of *I*_K(M)_ density elicited in response to membrane depolarization from −50 to −10 mV (indicated in the upper part) were obtained in the absence (a) and presence of 10 µM (b) and 30 µM (c) HBA. In (**B**), the time courses of the relative blocking of deactivating *I*_K(M)_-density trajectory by 10 µM HBA (1) and 30 µM HBA (2) were well fitted by single exponential with values of 19.0 and 12.9 ms, respectively (indicated by each smooth line). The relative block (i.e., (I_control_-I_HBA_)/I_control_) was evaluated by dividing the HBA-sensitive current density by the current density obtained in the control. In (**C**), the reciprocal time constant (i.e., 1/τ) of relative blocking versus the HBA concentration was plotted. Data points appearing in open circles were well fitted by a linear regression, indicating that there was a molecularity of one. According to the binding scheme described in the Materials and Methods Section, the blocking (*k*_+1_^*^) and unblocking (*k*_-1_) rate constants for HBA-induced blocking of *I*_K(M)_ were calculated to be 0.00235 ms^−1^µM^−1^ and 0.028 ms^-1^, respectively. Mean ± SEM (*n* = 9–11 for each point).

To provide a quantitative estimate for HBA-induced blocking of *I*_K(M)_, the time constants for the relative blocking of deactivating *I*_K(M)_ (i.e., (I_control_-I_HBA_)/I_control_) observed in these cells were further analyzed. Initially, the decaying time courses of relative blocking in the presence of different concentrations of HBA were fitted using a single-exponential function. The concentration dependence of *I*_K(M)_ decay elicited by membrane depolarization is illustrated in Figure 4A,B. It is clear from these results that the presence of HBA produced a concentration-dependent increase in the rate constant (1/τ) of relative blocking. For example, as cells were depolarized from −50 to −10 mV for 1 s, the decaying time constants (τ) of the *I*_K(M)_ relative block obtained in the presence of 10 and 30 µM were appropriately fitted with a single-exponential function with values of 18.5 ± 1.5 ms (*n* = 12) and 10.1 ± 1.2 ms (*n* = 11), respectively. Based on the first-order reaction scheme detailed in the Materials and Methods Section, the relationship between 1/τ and [B] was also found to be linear, with a correlation coefficient of 0.95 (Figure 4C). The blocking and unblocking rate constants for HBA-induced inhibition of *I*_K(M)_ were thus estimated to be 0.00235 ms^−1^µM^−1^ and 0.028 ms^−1^, respectively; consequently, the value of the dissociation constant (K_D_ = *k*_-1_/*k*_+1_^*^) turned out to be 11.9 µM, a value that is comparable with the IC_50_ value needed for HBA-mediated blocking of *I*_K(M)_ as described above.

### 2.5. Effect of HBA-Induced Blocking of I_K(M)_ Density Recorded from GH_3_ Cells

**Figure 5:**
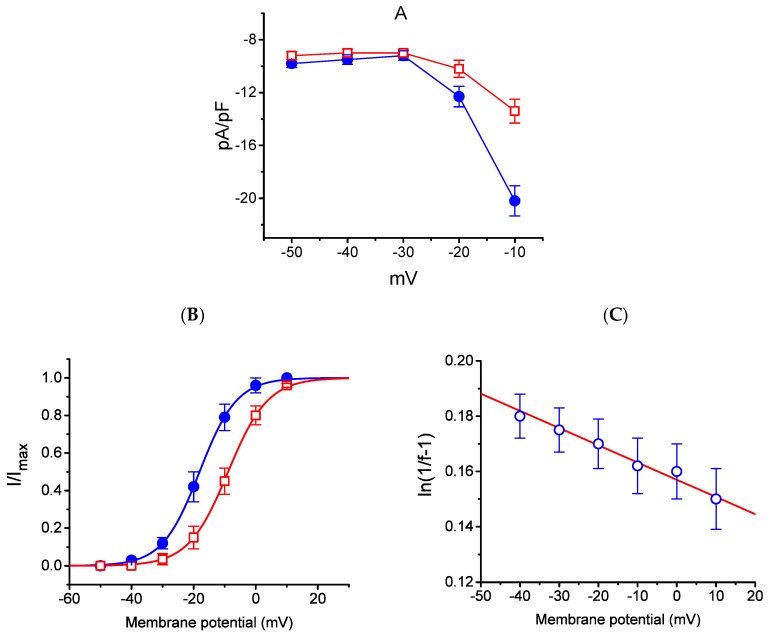
Effect of HBA on the average current density–voltage relations of *I*_K(M)_ density (**A**), the steady-state activation curve of *I*_K(M)_ (**B**), and the voltage-dependence of HBA-induced blocking of the current (**C**) in GH_3_ cells. Cells were bathed in high-K^+^, Ca^2+^-free solution and the pipette was filled with K^+^-containing solution. Once the whole-cell model was established, the examined cell was held at −50 mV and various voltages ranging between −50 and +10 mV were applied. (A) The relationship between *I*_K(M)_ density and membrane potential obtained in the presence (● blue) and presence (□ red) of 10 µM HBA (mean ± SEM; *n* = 9 for each point). (**B**) The activation curve of *I*_K(M)_ in the absence (● blue) and presence (□ red) of 10 µM HBA (mean ± SEM; *n* = 11 for each point). The smooth curves obtained with or without the addition of HBA were least-squares fitted by a Boltzmann function as detailed in the Materials and Methods Section. (**C**) Voltage dependence for HBA-induced blocking of *I*_K__(M)_ in GH_3_ cells (mean ± SEM; *n* = 10–11 for each point). The line overlaid onto the data was fitted using the equation as detailed in the Materials and Methods Section. The Kd value (the equilibrium constant at 0 mV) and δ (the relative electrical distance of the blocking site in the transmembrane electrical field) were estimated to be 11.7 µM and 0.016, respectively.

The effect of HBA on the *I*_K(M)_ elicited in response to different pulses from a holding potential of −50 mV was further studied. In this set of experiments, cells were bathed in a high-K^+^, Ca^2+^-free solution, and the recording pipette was filled with a K^+^-containing solution. Once the whole-cell model was established, the depolarizing pulses from −50 mV to various potentials ranging between −50 and +10 mV were delivered to the cells. The current density–voltage relationship for the inhibition effect of HBA (10 µM) on *I*_K(M)_ density is illustrated in Figure 5A, while the steady-state activation curve of *I*_K(M)_ obtained with or without the addition of HBA (10 µM) is shown in Figure 5B. The activation curve obtained in the absence or presence of HBA was plotted against the test potential and least-squares fitted by the Boltzmann equation as detailed in the Materials and Methods Section. In the control (i.e., in the absence of HBA), *V*_1/2_ = −18.1 ± 0.8 mV and *q* = 4.1 ± 0.4 *e* (*n* = 11), whereas during cell exposure to HBA (10 µM), *V*_1/2_ = −8.9 ± 0.5 mV and *q* = 3.8 ± 0.4 *e* (*n* = 11). As such, the addition of HBA not only produced a reduction in *I*_K(M)_ density, but also shifted the steady-state activation curve of the current in a rightward direction by approximately 9 mV. However, a minimal change in the estimated *q* value of the curve was detected during cell exposure to HBA, although the translocation of around 4 *e* across the electric field tends to be responsible for the voltage dependence of *I*_K(M)_ activation in GH_3_ cells. Moreover, the reversal potential of *I*_K(M)_ obtained in the absence and presence of 10 µM HBA did not differ (0 ± 2 mV (in the control) versus 0 ± 3 mV (in the presence of HBA), *n* = 7, *p* > 0.05). Consequently, the voltage-dependence of *I*_K(M)_ in these cells was virtually modified in the presence of HBA; however, the movement through the electrical field in response to different membrane potentials did not differ. The voltage-dependent relation of HBA-induced blocking was further determined using the equation as detailed in the Materials and Methods Section [17]. The estimated values of Kd (i.e., the equilibrium constant at 0 mV) and δ (i.e., the relative electrical distance of the blocking site in the transmembrane electrical field from outside) required for the inhibitory effect on *I*_K(M)_ were 11. 7 µM and 0.016, respectively (Figure 5C).

### 2.6. Effects of HBA on the Densities of I_K(erg)_ and I_K(DR)_ in GH_3_ Cells

**Figure 6:**
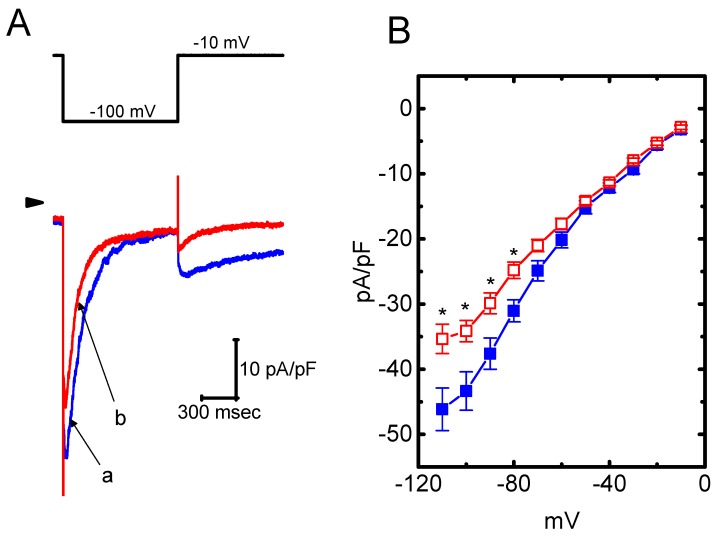

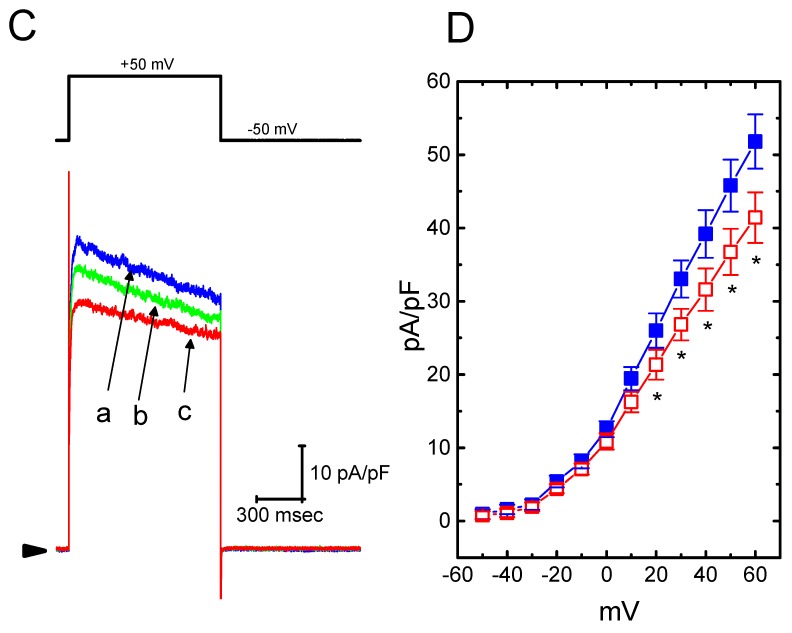
Effect of HBA on the densities of *I*_K(erg)_ and *I*_K(DR)_ recorded from GH_3_ cells. For the recording of *I*_K(erg)_ density, cells were bathed in high-K^+^, Ca^2+^-free solution, and to record the *I*_K(DR)_ density, cells were bathed in Ca^2+^-free Tyrode’s solution containing 1 µM tetrodotoxin. The recording pipette was filled with K^+^-containing solution. (**A**) Superimposed traces of *I*_K(erg)_ density elicited by membrane hyperpolarization (indicated in the upper part). The arrowhead shows the zero current level. (a) control; (b) 10 µM HBA. (**B**) Average current density–voltage relationships of *I*_K(erg)_ in the absence (■ blue) and presence (□ red) of 10 µM HBA (mean ± SEM; *n* = 9 for each point). Current density was obtained at the beginning of hyperpolarizing potentials from −10 mV to different voltages ranging between −110 and −10 mV. (**C**) Superimposed traces of *I*_K(DR__)_ density obtained in the absence (a) and presence of 3 µM HBA (b) and 10 µM HBA (c). The upper part indicates the voltage protocol used, and the arrowhead is the zero current level. (**D**) Average current density–voltage relationships of *I*_K(DR)_ taken with or without the addition of 10 µM HBA (mean ± SEM; *n* = 11 for each point). The *I*_K(DR)_ density was taken at the end of each depolarizing pulse. Asterisks shown in (B) and (D) indicate a significant difference from the controls taken from the same voltage level (*p* < 0.05). Note that, in comparison, the presence of HBA (10 µM) slightly suppressed the density of *I*_K(erg)_ or *I*_K(DR)_ elicited throughout the entire voltage-clamp ranges examined.

The presence of HBA can modify the amplitude and gating of *I*_K(M)_ observed in the GH_3_ cells. Whether other types of K^+^ currents (e.g., *I*_K(erg)_ or *I*_K(DR)_) enriched in these cells could be perturbed by HBA was further investigated in the current study. In order to measure *I*_K(erg)_ density, cells were bathed in high-K^+^, Ca^2+^-free solution, while to record the *I*_K(DR)_ density, they were bathed in Ca^2+^-free Tyrode’s solution. The recording pipettes used in these experiments were filled with K^+^-containing solution. As shown in Figure 6A,B, the addition of HBA (10 µM) slightly suppressed the amplitude of deactivating *I*_K(erg)_ density elicited by the entire voltage-clamp range. For example, as the examined cells were hyperpolarized from −10 to −100 mV, exposure to 10 µM HBA significantly decreased the *I*_K(erg)_ density from 43.3 ± 11 to 34.2 ± 9 pA/pF (*n* = 11, *p* < 0.05), although at a concentration of 3 µM, it produced a minimal effect on *I*_K(erg)_ density. Similarly, the *I*_K(DR)_ elicited by membrane depolarization from −50 to +50 mV was mildly suppressed by HBA (10 µM) (Figure 6C,D). For example, as cells were exposed to 10 µM HBA, the *I*_K(DR)_ density was significantly decreased to 36.7 ± 8.2 pA/pF from a control value of 45.8 ± 8.6 pA/pF (*n* = 11, *p* < 0.05); however, no evident change in the inactivation time course of *I*_K(DR)_ in response to membrane depolarization was demonstrated in its presence. After washout of the agent, the current amplitude returned to 46.4 ± 8.1 pA/pF (*n* = 9, *p* < 0.05). Therefore, these results showed that the density of either *I*_K(erg)_ or *I*_K(DR)_ in GH_3_ cells appeared to be relatively resistant to suppression by HBA.

### 2.7. Suppressive Effect of HBA on I_Ca,L_ Density Recorded from GH_3_ Cells

**Figure 7:**
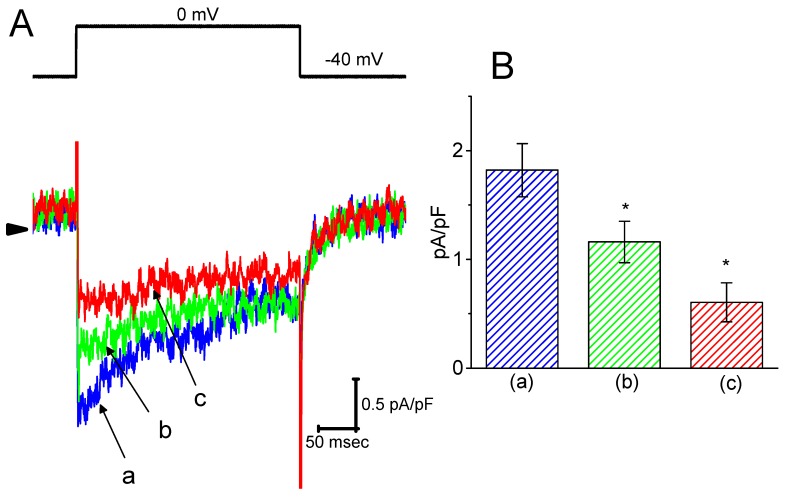
The inhibitory effect of HBA on ICa,L density in GH3 cells. In this set of experiments, cells were immersed in normal Tyrode’s solution containing 1.8 mM CaCl_2_, 1 µM tetrodotoxin, and 10 mM tetraethylammonium chloride, the composition of which is described in the Materials and Methods Section. The recording pipette was filled with a Cs^+^-containing solution. (**A**) Original traces of *I*_Ca,L_ density were elicited by membrane depolarization (indicated in the upper part). The arrowhead shows the zero current level. (a) control; (b) 3 µM HBA; and (c) 10 µM HBA. (**B**) Summary bar graph showing the effect of HBA on the peak density of *I*_Ca,L_ (mean ± SEM; *n* = 12 for each bar). The current density was measured at the beginning of each brief depolarizing pulse from −40 to 0 mV. (a) control; (b) 3 µM HBA; (c) 10 µM HBA. *p < 0.05 vs. the control.

It was further studied whether *I*_Ca,L_ density in GH_3_ cells could be modified during exposure to HBA. During these recordings, cells were bathed in normal Tyrode’s solution containing 1.8 mM CaCl_2_, 1 µM tetrodotoxin, and 10 mM tetraethylammonium chloride, and then the pipette was filled with a Cs^+^-containing solution. Tetrodotoxin is a potent blocker of *I*_Na_, while tetraethylammonium chloride is a non-specific K^+^ current blocker. Once the whole-cell mode was firmly established, a membrane depolarization from −40 to 0 mV with a duration of 300 ms was delivered to evoke *I*_Ca,L_ in these cells. As shown in Figure 7, as cells were exposed to HBA, the peak amplitude of *I*_Ca,L_ was progressively decreased. For example, as a depolarizing pulse from −40 to 0 mV was applied, the addition of HBA (10 µM) markedly decreased the peak *I*_Ca,L_ density from 1.8 ± 0.06 to 0.6 ± 0.03 pA/pF (*n* = 12, *p* < 0.05). The density of Ca^2+^-activated nonselective cationic current obtained following the return of the depolarizing pulse to the holding potential [18] was also concomitantly reduced from 0.46 ± 0.03 to 0.41 ± 0.02 pA/pF (*n* = 12, *p* < 0.05). Similar observations regarding the effect on peak *I*_Ca,L_ were obtained in the presence of GAS (10 µM). After washout of the drug, the *I*_Ca,L_ amplitude was returned to 1.3 ± 0.04 pA/pF (*n* = 11, *p* < 0.05). Therefore, it is conceivable that HBA and GAS exert a depressive action on *I*_Ca,L_ density in GH_3_ cells.

### 2.8. Lack of HBA Effect on I_Na_ Density in GH_3_ Cells

**Figure 8:**
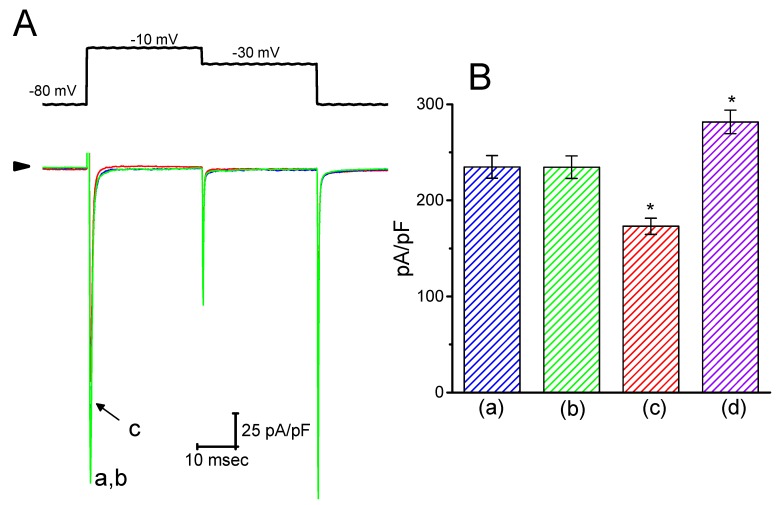
Effect of HBA on *I*_Na_ density recorded in GH_3_ cells. Cells were bathed in Ca^2+^-free Tyrode’s solution that contained 10 mM tetraethylammonium chloride and 0.5 mM CdCl_2_. For the recordings, the pipette was filled with Cs^+^-containing solution. (**A**) Superimposed traces of *I*_Na_ density elicited by brief depolarizing pulses (indicated in the upper part). The arrowhead indicates the zero current level. (a) control; (b) 30 µM HBA; (c) 30 µM HBA plus 10 µM A-887826. (**B**) Summary of the data showing the effects of HBA, HBA plus A-887826, and HBA plus orthovanadate on the peak density of *I*_Na_ (mean ± SEM; *n* = 11 for each bar). The peak density of *I*_Na_ was measured at the beginning of each brief depolarizing pulse from −80 to −10 mV. (a) control; (b) 30 µM HBA; (c) 30 µM HBA plus 10 µM A-887826; (d) 30 µM HBA plus 10 µM orthovanadate. *p < 0.05 vs. the control.

Earlier reports have revealed that HBA or GAS might exert anti-epileptic actions [5,7]. Blocking of *I*_Na_ was reported to have a protective role in hypoxia-induced injury and neuronal cell death [19]. For these reasons, it was further examined whether these agents might elicit any modifications on the amplitude or gating of *I*_Na_ in electrically excitable cells (e.g., GH_3_ cells). In this set of experiments, the cells were bathed in Ca^2+^-free Tyrode’s solution containing 10 mM tetraethylammonium chloride and 1 µM CdCl_2_, and the pipette was filled with a Cs^+^-containing solution. As shown in Figure 8, when the cells were exposed to 30 µM HBA, the amplitude of *I*_Na_ was not modified (235 ± 12 pA/pF (control) versus 235 ± 12 pA/pF (in the presence of 30 µM HBA), *n* = 11, *p* > 0.05). Similar results were obtained in the presence of 30 µM GAS. However, in the continued presence of 30 µM HBA, the addition of 10 µM A-887826, an inhibitor of the Na_V_1.8 channel [20], was found to decrease the amplitude of *I*_Na_ density in GH_3_ cells, as evidenced by a significant reduction in peak *I*_Na_ to 173 ± 8 pA/pF (*n* = 11, *p* < 0.05). Moreover, the subsequent application of orthovanadate (10 µM) in the presence of HBA increased the peak *I*_Na_ to 282 ± 12 pA/pF (*n* = 11, *p* < 0.05); however, neither the activation nor the inactivation time course of *I*_Na_ were changed. Orthovanadate has been previously reported to enhance the peak amplitude of cardiac *I*_Na_ [21]. Therefore, rather than having an inhibitory effect on *I*_Ca,L_ density, neither HBA nor GAS was capable of altering the amplitude or gating of peak *I*_Na_ density, even though they were previously revealed to exert anti-epileptic actions.

### 2.9. Suppressive Effect of HBA on I_K(M)_ Density in Hippocampal mHippoE-14 Neurons

**Figure 9:**
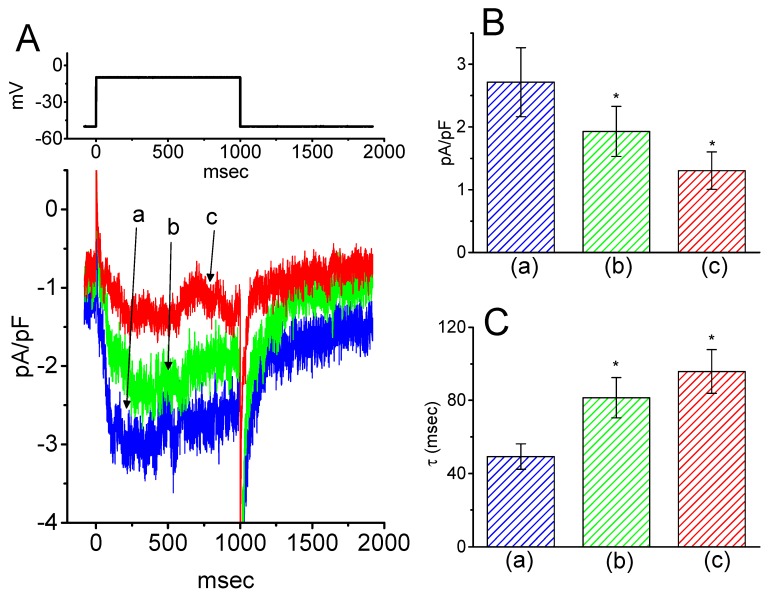
Inhibitory effect of HBA on *I*_K(M)_ density in hippocampal mHippoE-14 neurons. In this set of recordings, experimental conditions similar to those for the studies in GH_3_ cells described above were applied. (**A**) Superimposed traces of *I*_K(M)_ density elicited by membrane depolarization from −50 to −10 mV (indicated in the upper part). (a) control; (b) 3 µM HBA; (c) 10 µM HBA. In (**B**) and (**C**), the summary bar graphs show the effects of HBA on the density and τ_act_ of *I*_K(M)_, respectively (mean ± SEM; *n* = 9 for each bar). (a) control; (b) 3 µM HBA; (c) 10 µM HBA. *p < 0.05 vs. the controls. Note that, indistinguishable from the experimental results taken from GH_3_ cells as described above, the presence of HBA exerts an inhibitory effect on *I*_K(M__)_ density along with a conceivable slowing in the activation time course of the current density in these neurons.

Next, we tested whether *I*_K(M)_ in central neurons (e.g., hippocampal neurons) could be influenced by HBA. Previous reports have demonstrated the presence of *I*_K(M)_ density in the hippocampus [22]. The *I*_K(M)_ density identified in the hippocampal mHippoE-14 neurons appeared to be smaller than that in the GH_3_ cells. As depicted in Figure 9, as cells were exposed to different HBA concentrations, the density of *I*_K(M)_ was progressively decreased in response to the depolarizing pulse. Concomitant with this, a clear slowing in the activation time course of *I*_K(M)_ density was also observed. Furthermore, in the continued presence of 10 µM HBA, the subsequent addition of ML-213 (10 µM), an activator of *I*_K(M)_ [23], was effective at attenuating HBA-induced inhibition of *I*_K(M)_ density. These experimental results therefore indicate that HBA-induced inhibition of *I*_K(M)_ density tends to be indistinguishable from those described above in GH_3_ cells. 

## 3. Discussion

In the current study, two structurally related phenolic compounds, HBA and GAS, were evaluated for their possible effects on different types of ionic currents in the pituitary cells and hippocampal neurons. The important findings of the current study were as follows: first, in pituitary GH_3_ lactotrophs, HBA, and GAS suppressed the density of *I*_K(M)_ in a time- and concentration-dependent manner. Second, HBA-induced inhibition of *I*_K(M)_ density was associated with a slowing of the activation time course of *I*_K(M)_, while GAS-mediated inhibition was associated with the prolonging of the activation time course of the current. Third, HBA shifted the steady-state activation curve of *I*_K(M)_ toward a depolarized potential with no change in the gating charge of the current. Fourth, this agent slightly suppressed the density of *I*_K(erg)_ and *I*_K(DR)_. Fifth, it inhibited the density of *I*_Ca,L_, while no discernible change in peak *I*_Na_ density was detected in its presence. Sixth, in hippocampal mHippoE-14 neurons, the addition of HBA or GAS effectively inhibited the *I*_K(M)_ density. Therefore, because GAS can penetrate through the blood–brain barrier, different types of ionic currents, particularly low-threshold *I*_K(M)_ with slowly activating and deactivating properties, may be a relevant ‘target’ for the regulatory actions of these agents in neuroendocrine or central neurons, if similar in vivo findings occur.

The IC_50_ value of HBA or GAS needed to inhibit *I*_K(M)_ as observed in the GH_3_ cells were estimated to be 12.1 and 19.4 µM, respectively; this indicates the higher potency of HBA on the suppression of *I*_K(M)_. The peak concentrations of HBA in rat CSF and plasma after gastrodigenin (HBA) administration were previously reported to reach around 0.55 mg/mL (4.4 µM) and 0.47 (3.8 µM) µg/mL, respectively [24]. The present study also revealed the ability of this compound to shift the steady-state activation curve of *I*_K(M)_ to a depolarized potential, despite no conceivable change in the gating charge of the curve in its presence. Additionally, voltage-dependent blocking by HBA of *I*_K(M)_ with K_d_ (apparent affinity constant) or δ (fractional electrical distance) values of 11.7 µM and 0.016, respectively, were also further estimated in the current study. Therefore, it is important to point out that although the detailed ionic mechanisms of HBA action are still to be determined, modifications in the density and gating of *I*_K(M)_ during its exposure, as presented in the current study, were rapid and sensitive to the preexisting resting potential, the concentrations achieved, or both [14].

Blocking of *I*_K(M)_ density caused by HBA, not by GAS, does not tend to be instantaneous but instead develops over time after channels are opened upon abrupt membrane depolarization, which produces a significant decline in the slowly activating phase of the current (i.e., the activation time course). At the beginning, the depolarizing stimuli (i.e., dI/dt) are proportional to the number of channels available for activation. The trajectory of the *I*_K(M)_ density in the presence of HBA was noted to display a prolonged activation that may be explained by the binding model, which indicates that the opening of channels was delayed by binding of the HBA molecule to the open states. The deactivating time course of *I*_K(M)_ density in the presence of HBA or GAS also displayed a blunted peak together with increased decay, suggesting that the closing (i.e., deactivating process) of channels was considerably raised by unbinding of the HBA or GAS molecule.

In addition to the inhibition of *I*_K(M)_ amplitude, a notable elevation in the dI/dt and τ_act_ values of *I*_K(M)_ density were caused by the presence of GAS. These results imply that prior to channel activation, there might be a significant resting facilitation of the channel openness by GAS, but not by HBA. The effect of GAS on *I*_K(M)_ density might also exist in the closed states. A possible explanation for such a discrepancy in the influence on the activation time course of *I*_K(M)_ is that the sugar residue residing in the GAS molecule might facilitate the K_M_ channel openness upon membrane depolarization. The addition of ouabain (10 µM), which is recognized to be a Na^+^-pump inhibitor containing a sugar residue, was also observed to suppress *I*_K(M)_ density as well as enhance the value of τ_act_ in GH_3_ cells.

Intracellular zinc has been demonstrated to activate *I*_K(M)_ through the decreased dependence on phosphatidylinositol 4,5-bisphosphate [25]. HBA was also recently reported to exert anti-zinc toxicity in astrocytes and neurons [26]. However, in the present study, further addition of zinc chloride (1 mM) in the presence of HBA or GAS was unable to attenuate their effective inhibition of *I*_K(M)_ density in GH_3_ cells. The inhibitory effect of HBA or GAS on *I*_K(M)_ density is unlikely to be associated with changes in the level of intracellular inositol triphosphate.

Distinguishable from the inability of HBA to modify the peak density of *I*_Na_, the present results showed that the addition of HBA effectively suppressed the peak *I*_Ca,L_ density in GH_3_ cells, with no change in the overall I–V relationship of the current. The activity of *I*_Ca,L_ is well recognized to be enriched and functionally expressed in vascular smooth myocytes of different arteries including cerebral arteries. Therefore, to what extent HBA or GAS exerts beneficial effects on ischemic injuries in the brain [27,28] is connected, at least in part, through their inhibition of *I*_Ca,L_ density in excitable cells, warrants further investigation.

Minimal changes in the density or gating of *I*_Na_ in response to brief depolarizing pulses were observed in the presence of HBA or GAS. However, suppression by these agents of *I*_K(M)_ density was able to depolarize the resting potential, thereby causing excitable cells to become hypo-excitable because of the resting inactivation of *I*_Na_ in these cells. Therefore, it is possible that the antiepileptic actions exerted by these agents can be attributed to other mechanisms such as GABAergic system enhancement and inhibition of glutamate induced excitotoxicity [29,30].

There have been conflicting opinions on the role played by HBA and GAS and their potential neuropharmacological effects, since HBA could also exert pharmacological effects on the central nervous system itself by binding with the benzodiazepine (or GABA_A_) receptor in brain membranes [31]. However, in the present study, further application of flumazenil (10 µM), still in the presence of GAS or HBA, did not attenuate the inhibitory actions on *I*_K(M)_ density in GH_3_ cells. Flumazenil is known to be a benzodiazepine antagonist. The blocking of *I*_K(M)_ by HBA and GAS seen in GH_3_ and mHippoE-14 cells is not highly associated with their propensity to interact with benzodiazepine receptors. However, whether the disparate action of HBA and GAS on the τ_act_ value of *I*_K(M)_ density could be partly explained by their different neuropharmacological actions occurring in vivo, is worthy of further investigation. Moreover, based on the molecular mechanism reported herein, HBA and GAS may have potential as pharmacological probes that could be used for the characterization of K_M_ or Ca_V_ channel properties as well as in therapeutic practice.

## 4. Materials and Methods 

### 4.1. Drug and Solution Preparations

Gastrodin (GAS, gastrodine, p-hydroxymethylphenyl-β-D-glucopyranoside, 4-hydroxybenzyl alcohol-4-β-D-glucopyranoside, (2R,3S,4S,5R,6S)-2-(hydroxymethyl)-6-[4-(hydroxymethyl) phenoxy] oxane-3,4,5-triol, C_13_H_18_O_7_, PubChem CID: 115027), flumazenil, orthovanadate, ouabain, tetraethylammonium chloride, and tetrodotoxin were obtained from Sigma-Aldrich (St. Louis, MO, USA); gastrodigenin (p-hydroxybenzyl alcohol, 4-hydroxybenzyl alcohol, HBA) was purchased from Santa Cruz Biotechnology (Santa Cruz, CA, USA); and A-887826, flupirtine and ML-213 were obtained from Tocris Cookson (Bristol, UK). Unless stated otherwise, the culture media, L-glutamine, and trypsin were obtained from Invitrogen (Carlsbad, CA, USA). All other chemicals including CsCl, CdCl_2_, CsOH, and ZnCl_2_ were of the highest commercially available purity. The deionized water used throughout the experiments was made using a Milli-Q water purification system (APS Water Services, Inc., Van Nuys, CA, USA).

The composition of the bath solution (HEPES-buffered normal Tyrode’s solution) was 136 mM NaCl, 5.4 mM KCl, 1.8 mM CaCl_2_, 0.53 mM MgCl_2_, 5.5 mM glucose, and 5.5 mM HEPES-NaOH buffer, pH 7.4. To measure macroscopic K^+^ currents (e.g., *I*_K(M)_, *I*_K(erg)_, or *I*_K(DR)_) and preclude contamination of Cl^-^ currents, the path pipettes were filled with a solution containing 130 mM K-aspartate, 20 mM KCl, 1 mM KH_2_PO_4_, 1 mM MgCl_2_, 3 mM Na_2_ATP, 0.1 mM Na_2_GTP, 0.1 mM EGTA, and 5 mM HEPES-KOH buffer, pH 7.2. For the recordings of whole-cell *I*_K(M)_ or *I*_K(erg)_, a high K^+^-bathing solution consisting of 145 mM KCl, 0.53 mM MgCl_2_, and 5 mM HEPES-KOH buffer, pH 7.4 was used, whereas the recording pipette was filled with a solution containing 145 mM KCl, 2 mM MgCl_2_, and 5 mM HEPES-KOH buffer, pH 7.2. To measure *I*_Ca,L_ or *I*_Na_, the K^+^ ions inside the pipette solution were replaced with equimolar Cs^+^ ions, and the pH was then adjusted to 7.2 with CsOH. Each solution (e.g., the bath and pipette solutions) was filtered on the day of use with a disposable syringe filter with a 0.22 µm pore size (Millipore, Bedford, MA, USA).

### 4.2. Cell Preparations

GH_3_ pituitary tumor cells were obtained from the Bioresources Collection and Research Center ([BCRC-60015]; Hsinchu, Taiwan) and maintained in Ham’s F-12 medium supplemented with 15% horse serum (*v*/*v*), 2.5% fetal calf serum (*v*/*v*), and 2 mM L-glutamine in a humidified environment of 5% CO_2_/95% air. The embryonic mouse hippocampal cell line (mHippoE-14, CLU198) was obtained from Cedarlane CELLutions Biosystems, Inc. (Burlington, ON, Canada). Cells were grown in Dulbecco’s modified Eagle’s medium supplemented with 10% fetal bovine serum (*v*/*v*) and 2 mM L-glutamine [15]. The culture medium was changed every 2–3 days for the removal of non-adhering cells, and cells were passaged as they reached confluence.

### 4.3. Electrophysiological Measurements

Shortly before the experiments, the cells (i.e., GH_3_ cells and mHippoE-14 neurons) were dissociated, and an aliquot of cell suspension was transferred to a home-made recording chamber that was mounted on the stage of a DM-IL inverted microscope (Leica, Wetzlar, Germany), and then left to settle. The cells were immersed at room temperature (20–25 °C) in HEPES-buffered normal Tyrode’s solution, the composition of which is described above. For the recordings, patch electrodes were fabricated from borosilicate glass capillaries (#34500; Kimble Products, Vineland, NJ, USA) on a Narishige PP-830 puller (Narishige, Tokyo, Japan), then fire-polished with an MF-83 microforge (Narishige). The resistances existing between the standard pipette and the bathing solution ranged between 3 and 5 MΩ. Recordings of different types of ionic currents were measured in the whole-cell mode using the standard patch-clamp technique with either an RK-400 (Bio-Logic, Claix, France) or an Axopatch 200B patch-clamp amplifier (Molecular Devices, Sunnyvale, CA, USA) [32], which was interfaced via a Digidata 1440A to a PC running pCLAMP suite software (Molecular Devices). The liquid junction potentials were corrected shortly before seal formation was established.

### 4.4. Data Recording

The signals comprising both potential and current traces were stored online and sampled at 10 kHz using an ASUS VivoBook Flip-14 touchscreen laptop computer (TP412U; Taipei City, Taiwan), which was equipped with a Digidata 1440A interface (Molecular Devices) for analog-to-digital/digital-to-analog conversion. During the recordings, the latter device was driven by pCLAMP 10.7 software (Molecular Device) running on Windows 10 (Redmond, WA, USA), and the signals were simultaneously monitored on an MB169B+ monitor (ASUS, Taipei, Taiwan) through a USB type-C connection. When current signals were acquired, they were low-pass filtered at 2 kHz with a FL-4 four-pole Bessel filter (Dagan, Minneapolis, MN, USA) to minimize background noise. Through digital-to-analog conversion, various voltage-clamp profiles with either rectangular or ramp waveforms were created from pCLAMP and then applied to evaluate either the current versus voltage (I–V) relationship or the steady-state activation curve for the different types of ionic currents. As high-frequency stimuli needed to be delivered, an Astro-med Grass S88X dual output pulse stimulator was used (Grass Technologies, West Warwick, RI, USA). After the data were digitally collected, they were then analyzed using different analytical tools including the LabChart 7.0 program (AD Instruments, Castle Hill, Australia), 64-bit OriginPro 2016 (Microcal, Northampton, MA, USA), or custom-made macros built under Microsoft Excel 2013 (Redmond).

### 4.5. Concentration-Dependent Analyses of I_K(M)_

To evaluate the concentration-dependent effect of HBA or GAS on the amplitude of *I*_K(M)_, GH_3_ cells were immersed in high-K^+^, Ca^2+^-free solution. Each examined cell was clamped at −50 mV and a 1-s depolarizing pulse from −50 to −10 mV was delivered. Current amplitudes at the end of the depolarizing pulse from −50 to −10 mV were measured in the control and during cell exposure to different concentrations (0.3–300 µM) of HBA or GAS. The concentration required to suppress 50% of the *I*_K(M)_ amplitude was determined using a Hill function:

(1) $$\mathrm{Relative}\;\mathrm{amplitude}=\frac{\left(1-a\right)\times {\left[C\right]}^{-n_{H}}}{{\left[C\right]}^{-n_{H}}+{\mathrm{IC}}_{50}^{-n_{H}}}+a$$

where [C] represents the concentration of HBA or GAS; IC_50_ and n_H_ are the concentration required for a 50% inhibition and the Hill coefficient, respectively; maximal inhibition (i.e., 1-a) was also estimated in this equation. This equation converged reliably to produce the best-fit line and parameter estimates as shown in Figure 3.

### 4.6. State-Dependent Analyses

The inhibitory effect of GAS or HBA on the *I*_K(M)_ measured from pituitary GH_3_ cells may be explained by a state-dependent blocker that preferentially binds to the open state of the K_M_ channel. A minimal kinetic scheme was postulated and derived as follows:
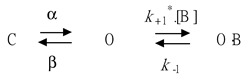
(2)
where α and β are the voltage-gated rate constants for the opening and closing of K^+^ channels; *k*_+1_^*^ and *k*_-1_ are the blocking and unblocking rate constants in the presence of HBA or GAS, respectively; C, O, and O B are the closed, open, and open-blocked states, respectively; and [B] is the HBA or GAS concentration applied. The blocking (i.e., on) and unblocking (i.e., off) rate constants, *k*_+1_^*^ and *k*_-1_, were determined from the time constants (τ) of the depolarization-elicited relative block (i.e., (I_control_-I_compound_)/I_control_) of deactivating *I*_K(M)_ obtained in different concentrations of HBA or GAS. These rate constants were then computed according to the following relationship:

(3) $$\frac{1}{\tau }=k_{+1}{}^{*}\times [B]+k_{-1}$$

where *k*_+1_* and *k*_-1_ are respectively derived from the slope and from the y-axis intercept at [B] = 0 of the linear regression interpolating the reciprocal time constants (1/τ) versus the HBA or GAS concentration, and [B] is the HBA or GAS concentration. Results from the blocker kinetic analysis are illustrated in Figure 4C.

### 4.7. Voltage-Dependent Analyses of HBA Effects on I_K(M)_

To determine the effect of HBA on the steady-state activation curve of *I*_K(M)_ in GH_3_ cells, the cells were bathed in a high-K^+^, Ca^2+^-free solution. Once the whole-cell mode was established, the examined cells were maintained at −50 mV and voltage pulses were then applied from −50 mV to various test potentials up to +10 mV, with a duration of 1 s; these were delivered by the pCLAMP 10.7 program (Molecular Devices) through digital-to-analog conversion. The activation curve of the tail *I*_K(M)_ obtained upon repolarization following each depolarizing pulse to the holding potential with or without the addition of HBA was appropriately fitted using the Boltzmann equation:

(4) $$\frac{I}{I_{max}}=\frac{1}{1+exp\left[-\left(V-V_{\frac{1}{2}}\right)qF/RT\right]}$$

where *I*_max_ is the maximal amplitude of deactivating *I*_K(M)_; *V* is the membrane potential in mV; *V*_1/2_ the voltage at which there is half-maximal activation of the current; *q* is the apparent gating charge (i.e., the charge across the membrane electrical field between closed and open conformations); R is the universal gas constant; F is Faraday’s constant; T is the absolute temperature; and F/RT = 0.04 mV-1. To determine the voltage dependence, the HBA-induced block on *I*_K(M)_ was assessed by fitting Equation (5) [17], taking the form:

(5) $$f=\frac{\left[B\right]}{\left[B\right]+K_{d}exp\left(\frac{-\delta FV}{RT}\right)}\;\ln\left(\frac{1}{f}-1\right)=ln\left(\frac{K_{d}}{\left[B\right]}\right)+\left(\frac{-\delta FV}{RT}\right)$$

where f is the fractional block (i.e., 1 – I_HBA_/I_control_); [B] is the HBA concentration; *V* is the membrane potential in mV; δ is the fractional electrical distance (i.e., the relative electrical distance of the blocking site in transmembrane electrical fields from outside); K_d_ is the affinity at the reference voltage (0 mV); and F, R and T are as described above.

### 4.8. Statistical Analyses

The linear or nonlinear (e.g., sigmoidal (dose-response or steady-state activation curve) or exponential functions) least-squares fitting procedures were implemented using either the Solver add-in bundled with Microsoft Excel 2013 (Redmond) or OriginPro 2016 (OriginLab, Northampton, MA, USA). The data are presented as the mean ± standard error of the mean (SEM) with sample sizes (n) indicating the number of cells from which the experimental results were collected. The error bars in all figures are plotted as SEM. A paired or unpaired Student’s *t*-test was used for the comparison of paired groups, and one-way analysis of variance (ANOVA) followed by a post-hoc Fisher’s least-significance difference test for multiple comparisons was implemented for the statistical evaluation of the differences between means. However, we used a non-parametric Kruskal–Wallis test if the assumption of normality underlying ANOVA could be violated. Statistical analyses were performed using IBM SPSS version 20.0 (IBM Corp., Armonk, NY, USA). A difference with a *p* value < 0.05 was considered to be statistically significant, unless otherwise stated.

## 5. Conclusions

Our present study provides the first electrophysiological evidence to indicate that HBA or GAS, the bioactive ingredients from GEB, exerted inhibitory actions on the M-type K^+^ current density in a concentration-dependent manner in pituitary GH_3_ cells. The presence of HBA also slightly suppressed the density of the erg-mediated or delayed-rectifier K^+^ current in GH_3_ cells. Apart from the K^+^ currents, HBA attenuated the density of the L-type Ca^2+^ current at the same concentration, although neither GAS nor HBA modified the voltage-gated Na^+^ current density. Moreover, in hippocampal mHippoE-14 neurons, HBA was effective at inhibiting *I*_K(M)_ density as well as slowing the activation time course, which was similar to those findings observed in GH3 cells. The modulation of ionic current densities demonstrated by the present study conceivably represents a part of the fundamentally molecular mechanisms through which HBA and GAS can exert pharmacologic properties and functional influence in various neurologic disorders.

## Abbreviations

Ca_V_	Calcium channel
GAS	Gastrodin
GEB	Gastrodia elata Blume
HBA	p-hydroxybenzyl alcohol
I–V	Current versus voltage
IC_50_	The concentration required for 50% inhibition
*I*_Ca,L_	L-type Ca^2+^ current
*I*_K(DR)_	Delayed-rectifier K^+^ current
*I*_K(erg)_	Erg-mediated K+ current
*I*_K(M)_	M-type K^+^ current
*I*_Na_	Voltage-gated Na^+^ current
K_M_	M-type K^+^ channel
K_V_	Potassium channel
Na_V_	Sodium channel
SEM	Standard error of mean
τ_act_	Activation time constant
τ_deact_	Deactivation time constant
